# Natural history of liver fluke infection underpins epidemiological patterns of biliary cancer

**DOI:** 10.1073/pnas.2423536122

**Published:** 2025-10-10

**Authors:** Thomas Crellen, Francesca Vita, Chiara Braconi, Paiboon Sithithaworn, T. Déirdre Hollingsworth

**Affiliations:** ^a^Saw Swee Hock School of Public Health, National University of Singapore, Singapore 117549, Singapore; ^b^Big Data Institute, Li Ka Shing Centre for Health Information and Discovery, University of Oxford, Oxford OX3 7LF, United Kingdom; ^c^Nuffield Department of Medicine, Centre for Tropical Medicine and Global Health, University of Oxford, Oxford OX3 7LF, United Kingdom; ^d^School of Biodiversity One Health and Veterinary Medicine, University of Glasgow, Glasgow G12 8QQ, United Kingdom; ^e^School of Cancer Sciences, Wolfson Wohl Cancer Research Centre, University of Glasgow, Glasgow G61 1QH, United Kingdom; ^f^Department of Oncology, University of Turin, Turin 10043, Italy; ^g^Beatson West of Scotland Cancer Centre, Glasgow G12 0YN, United Kingdom; ^h^Cancer Research UK Scotland Cancer Centre, Glasgow G61 1BD, United Kingdom; ^i^Department of Parasitology, Faculty of Medicine, Khon Kaen University, Khon Kaen 40002, Thailand; ^j^Cholangiocarcinoma Research Institute, Khon Kaen University, Khon Kaen 40002, Thailand

**Keywords:** epidemiology, cancer evolution, parasitology, neglected tropical diseases

## Abstract

Pathogens are major drivers of cancer globally and the processes of infection and carcinogenesis unfold over decades making them difficult to observe in human or natural populations. We investigate these hidden dynamics for the foodborne trematode *Opisthorchis viverrini*, which is a primary cause of biliary cancer (cholangiocarcinoma) and infects 12 million people in Southeast Asia. In tumors from patients exposed to *O. viverrini*, we find that the earliest chromosomal amplifications carrying driver genes occurred at 30 y old on average, two to four decades before cancer diagnosis, and disproportionately contain *TP53*, *PTEN* and *FGFR2* genes. We then fitted transmission models to parasitological data from Thailand spanning 27 y (*n* = 11,517) finding that, for people born between 1960 and 1989, first exposure occurred at two years old and by 30 y individuals had been cumulatively infected with a median of 72 worms. Trematodes are long-lived and our analysis quantifies the average lifespan of *O. viverrini* as 13 y (90% credible interval [CrI] 7 to 26 y) within human hosts. The lifetime probability of diagnosis with cholangiocarcinoma is 4.9% (90% CrI 4.7 to 5.0%) given prior exposure to *O. viverrini*, which is fourteen-fold higher than in populations nonendemic for the parasite. We find strong evidence for a dramatic decline in parasite transmission from 1990 onward in Thailand, suggesting that the incidence of cholangiocarcinoma will decline over the coming decades. Our study demonstrates how pathogen exposure drives patterns of cancer within a population and provides evidence for public health and therapeutic interventions.

## Significance

Infestation with parasitic worms can cause cancer in humans, though this decades-long process is challenging to study. Here, we show how liver fluke ecology determines the epidemiology of cholangiocarcinoma (bile duct cancer) in Thailand. We first characterize the natural history of infection. People are exposed by consuming raw freshwater fish before ten years of age, and worms survive for 13 y inside humans. The earliest driver mutations emerge at 30 y old, which is three decades before cancer diagnosis and provides a window for preventative treatment. We calculate that 5% of infected people acquire cholangiocarcinoma, which is fourteen times higher than populations without liver fluke. Lower parasite transmission after 1990 suggests that cholangiocarcinoma incidence will decline in the future.

Infectious organisms are a major contributor to the burden of human cancers globally (1), thus challenging the classic distinction between “communicable” and “noncommunicable” diseases. The epidemiology of pathogen-induced cancers includes the transmission of the infectious agent, which is a dynamic population-level process, plus the resulting within-host pathology that drives carcinogenesis (2). These processes are complex, unfold over many years, and are unlikely to be co-observed in a single prospective cohort (3).

Of the eleven pathogens recognized as direct human carcinogens; three are parasitic trematodes.^*^,^†^ Infection with the foodborne liver flukes *Opisthorchis viverrini* and *Clonorchis sinensis* cause biliary cancer (cholangiocarcinoma), while the waterborne blood fluke *Schistosoma haematobium* causes cancer of the bladder (squamous cell carcinoma). The pathology arising from infection with parasitic worms is typically chronic as definitive hosts rarely develop protective immunity to reinfection, making it difficult to quantify the impact of any single helminth species over decades of exposure and in populations with coinfections (4, 5).

Our study focuses on the liver fluke *O. viverrini*, which is acquired by eating raw freshwater fish; a traditional component of the diet in regions of Southeast Asia (6). The fluke has a complex lifecycle, which involves asexual reproduction within freshwater snails and encysts as a mammalian-infective stage (metacercariae) in cyprinid fish. Despite public health programs to control the parasite, an estimated 12 million people were infected with *O. viverrini* across Thailand, Lao PDR, Cambodia, and Vietnam in 2018 (7). While the prevalence has shown gradual declines in Thailand due to parasite control programs during the second half of the twentieth century (8), progress has recently slowed (9, 10). In Cambodia and Lao PDR, by contrast, there is evidence of increased transmission over the past two decades (7, 11). Liver fluke-endemic countries have the highest incidence of cholangiocarcinoma globally and cases of hepatic cancers in these regions are disproportionately attributable to cholangiocarcinoma, rather than hepatocellular carcinoma (12, 13). The mechanisms through which flukes induce cholangiocarcinoma is a combination of mechanical damage, inflammation of the biliary epithelium, and the secretion of proteins; in particular, the peptide granulin (14).

Given the poor prognosis for cholangiocarcinoma (15) and the preventable nature of parasite infection, there is a strong motivation to understand the link between liver fluke exposure and carcinogenesis in humans (16). Prior to the onset of driver mutations, anthelmintic treatment and reducing parasite exposure should be prioritized as public health interventions, whereas after the onset of irreversible malignancies the priority for interventions shifts to ultrasound screening for liver pathology and early referral for hepatobiliary surgery (17).

This study infers the timings of driver mutations for fluke-induced biliary cancer using computational methods that characterize the evolution of tumors from a single biopsy (18, 19). We then define the age of first exposure to the parasite by fitting dynamic transmission models to parasitological survey data from Thailand. Finally, we estimate the lifetime probability of diagnosis with cholangiocarcinoma given infection with *O. viverrini*. By combining evolutionary cancer genomics with epidemiological analysis, we obtain unique insights into the relationship between pathogen exposure and tumorigenesis, with implications for evidence-based disease control.

## Results

### Cholangiocarcinoma Tumor Genomes.

We obtained paired tumor and normal whole-genome sequences from cholangiocarcinoma patients who were previously infected with liver fluke and treated at a large public hospital in Northeast Thailand (20). We used a bioinformatics pipeline to call somatic single nucleotide variants (SNVs), copy number alterations, and inferred the clonal status of these mutations (*Materials and Methods*). The age of the patients at surgery ranged from 37 to 79 y (median 57 y), 50% of patients were female, and all were born prior to 1980 (20). After mapping reads, variant calling, and filtering (*Materials and Methods*) we obtained 2,349 to 27,821 (median 10,360) SNVs and 268 to 14,230 (median 1,382) somatic indels per tumor. The overall ploidy (chromosomal copies) per tumor ranged from 1.2 to 3.7 (median 2.0). Cancer genomic and patient data are summarized in *SI Appendix*, Table S1.

### Evolution of Cholangiocarcinoma Tumors.

**Fig. 1.**
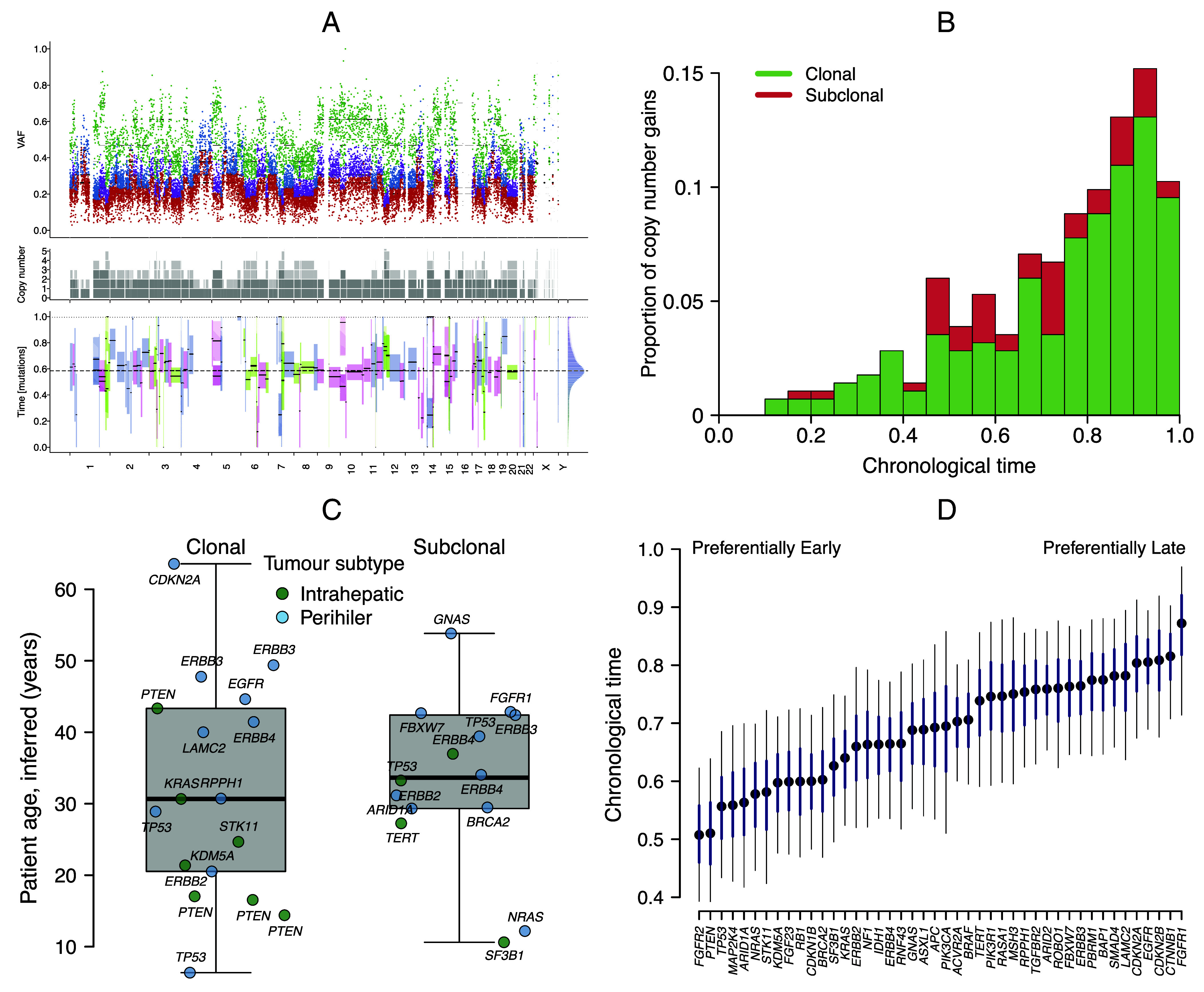
Evolution of cholangiocarcinoma tumors. (*A*) Subclonal lineage reconstruction for tumor CCA_TH_19. The *Upper* plot shows the variant allele frequency (VAF) for SNVs which are colored by clonal state; clonal (early) = green, clonal (late) = purple, clonal (unknown) = blue, and subclonal = red (19). The *Middle* plot shows the inferred copy number frequency (ploidy) by chromosome. The *Lower* plot shows the inferred timings of copy number gains. For this tumor, the timings of copy number gains are correlated across chromosomes, indicating a whole-genome duplication event at a “chronological time” of 0.6. (*B*) Histogram showing the timing of 287 clonal and subclonal amplification events for 44 driver mutations in 17 cholangiocarcinoma tumors (23). (*C*) The earliest amplified clonal and subclonal driver mutations in cholangiocarcinoma (CCA) tumors, where the timing is estimated using CpG>TpG mutations multiplied by the patient age at surgery (20) to give the inferred age. Each point represents the first amplified driver gene per patient, labeled with the gene name, and the points are colored by tumor anatomical subtype (intrahepatic or perihilar). The overlaid box and whisker plots show the median and interquartile range. (*D*) Estimates of the chronological time of amplification by gene. Results are shown from a generalized linear model applied to estimates from AmplificationTimeR (23). The black points give the posterior median, the thick blue line gives the 50% credible interval and the thin black line the 95% credible interval.

We estimated the timing of driver mutations in 43 genes implicated in cholangiocarcinoma development (21, 22) using evolutionary models that time chromosomal amplifications using the number of accumulated SNVs on different chromosomal copies. We first assessed whether any tumors had been subjected to whole-genome duplication events based on the correlated timings of chromosomal amplifications throughout the genome (19) and concluded that this had occurred in three tumors (Fig. 1*A*). Across all tumors, the copy number was disproportionately higher in chromosomes 7 and 17. In 17/22 tumors with sufficient ploidy and tumor purity, we inferred the timing of focal chromosomal amplifications in potential driver genes for cholangiocarcinoma. Overall the majority of amplifications (166/287; 58%) occurred later in “chronological time” (0.75 or later), with a smaller proportion (46/287; 16%) occurring earlier in tumorigenesis (before 0.5), as shown in Fig. 1*B*.

### Timing of Amplified Driver Mutations.

We determined the earliest amplified driver gene for each tumor sample, and classified these as either “clonal” (occurring on the most recent common cell lineage of the tumor) or “subclonal” (a subsequent clonal expansion within the tumor that has not risen to fixation) (24). To calculate the age at which these amplifications occurred in patients, we scaled the chronological time estimate with the patient’s age at surgery (*Materials and Methods*). The first clonal amplification of driver genes occurred at a median age of 30 y and with an interquartile range (IQR) of 20 to 43 y (Fig. 1*C*). The first subclonal amplifications occurred at a median age of 33 y (IQR 29 to 41 y). A variety of genes were the earliest amplified, with the most frequently occurring being the tumor suppressor *PTEN* in the clonal lineages (earliest in four tumors and amplified in 7/17 tumors), while the tumor suppressor *TP53* and the protein kinase *ERBB4* were the most common in subclonal lineages (earliest in two tumors and amplified in 7/17 and 6/17 tumors respectively) see Fig. 1*B*. Our timing of somatic events uses C>T mutations at CpG sites (CpG>TpG; also known as single base substitution signature 1 [SBS1]) which has clock-like properties as the mutational load correlates with age in cancer patients (25). However, the CpG>TpG mutation rate may accelerate with age in cancerous cells (19). We therefore compared findings from the constant rate model above against alternative scenarios where the CpG>TpG mutation rate accelerates 2, 5, 8, or 12-fold during tumorigenesis (*SI Appendix*, Fig. S1). In a scenario where the CpG>TpG mutation rate accelerates fivefold over the lifetime of the patient, the age of earliest amplified driver genes for cholangiocarcinoma increases to 36 y on average (IQR 28 to 46 y).

To determine whether certain genes are disproportionately likely to be amplified early or late within the lifespan of the tumor, we applied a generalized linear model to the chronological age estimates for each of the amplification events, while controlling for host sex (female [reference] or male), tumor anatomical subtype (intrahepatic [reference] or perihilar), and clonality (clonal [reference] or subclonal); see *Materials and Methods* and Eq. **2**. We restricted this analysis to amplification events with at least ten CpG>TpG SNVs, giving 271 amplification events from 17 tumors. The model estimates per gene are shown by chronological age in Fig. 1*D*. Overall, the driver genes which were disproportionately found to be amplified early were the fibroblast growth factor receptor *FGFR2*, *PTEN* and *TP53* (these genes were amplified in 7 tumors), while the tumor suppressor genes *BAP1* and *PBRM1* (both amplified in 5 tumors), and the receptor gene *FGFR1* (amplified in 4 tumors) were amplified later in tumorigenesis. The coefficients from the generalized linear model indicate that clonality did not affect gene amplification times (odds ratio [OR] 0.96; 0.74 to 1.27), although male patient sex (OR 1.23; 90% CrI 1.00 to 1.55) and perihilar anatomical subtype (OR 1.39; 90% CrI 1.12 to 1.71) were both associated with later timings of amplification events. Our findings add support to previous studies that have noted early clonal amplification of *TP53*, in particular, as a driver across a range of cancer types (19).

### Liver Fluke Transmission.

As fluke-induced pathology of the biliary tract is chronic, to understand the etiology of cholangiocarcinoma, it is necessary to consider prior exposure to the parasite at the individual or population level which can be estimated from historical parasitological surveys (2). Therefore, we collated epidemiological surveys from Thailand in which diagnostic observations of *O. viverrini* infection intensity (worm burdens or fecal egg counts) were available by host age. Our analysis uses data from 4,056 individuals obtained from seven surveys conducted between 1980 and 1989, prior to the onset of large-scale control programs against liver fluke (8), and 7,448 individuals from five surveys conducted between 1994 and 2017, which are summarized in Table 1 and *SI Appendix*, Fig. S2. The parasite prevalence by age in surveys pre- and postintervention are shown in *SI Appendix*, Figs. S3 and S4. We fitted a mechanistic parasite transmission model to these data where the parasite burden at a given age is the result of flukes infecting the host with an age-variable transmission rate, referred to as the “force of infection,” and flukes exiting the host due to the spontaneous death rate of adult worms (35). See *Materials and Methods* for model details and *SI Appendix*, Table S2 for all parameter values.

**Table 1.** Cross-sectional surveys analyzed in this study investigating the relationship between host age and *Opisthorchis viverrini* worm burden, fecal egg counts, or prevalence by fecal egg diagnostic (Fig. 2*A*)

Survey & method	Province	District	Year(s)	*n* ^*^	Age range
S1 Autopsy (26)	Khon Kaen	Multiple^†^	1982–1989	159	2 to 78
S2 Expulsion (27)	Khon Kaen	Ban Nam	1987	33	15 to 56
S3 Expulsion (28)	Kalasin	Huai Mek	1989	373	5 to 60
S4 fecal egg (29)	Khon Kaen	Chonnabot	1980	1,651	1 to 72
S5 fecal egg (30)	Multiple	Rural^‡^	1981–1983	433	1 to 65
S6 fecal egg (30)	Multiple	Urban^‡^	1981–1983	126	1 to 70
S7 Prevalence (31)	Khon Kaen	Nong Wai	1981	1,284	1 to 65
S8 fecal egg (8)	Multiple	Multiple	1994	65	1 to 70
S9 Prevalence (8)	Multiple	Multiple	1994	1,912	1 to 70
S10 Prevalence (32)	Ratchasima	Multiple	2010–2011	1,168	5 to 90
S11 Prevalence (33)	Multiple	Multiple	2013	3,916	15 to 70
S12 Prevalence (34)	Khon Kaen	Khon Kaen	2016–2017	387	11 to 91

Surveys were conducted in Northeast (N.E.) Thailand either before the onset of liver fluke control programs (S1 to S7; preintervention), or following a national control program in the early 1990s (S8 to S12; postintervention). Age range of the participants is shown in years.

^*^Sample size of human participants in survey.

^†^Autopsy cases were from N.E. Thailand, with the majority from Khon Kaen province.

^‡^Hospital-based study where patients from N.E. Thailand were recruited and classified as originating from either rural or urban communities.

### Exposure to Liver Fluke in Human Populations.

**Fig. 2.**
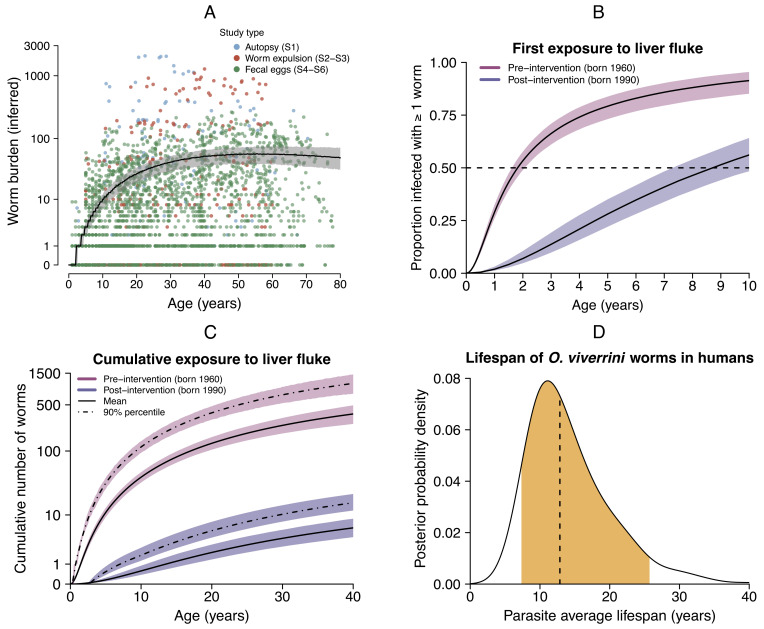
Epidemiological insights into the carcinogenic liver fluke *Opisthorchis viverrini* in Thailand. (*A*) Worm burdens of *O. viverrini* by participant age, inferred by a parasite transmission model from multiple surveys conducted between 1980 and 1989 prior to large-scale interventions. The data used for model fitting were counts of i) adult worms obtained from autopsy, or ii) worm expulsion, or iii) parasite eggs in feces (data shown from S1 to S6; see Table 1). Observed data were transformed into inferred worm burdens using Eqs. **3**, **8**, and **14**; see *Materials and Methods* and ref. 36. The fitted black line shows the simulated median worm burden by host age, with the shaded area giving the 90% prediction interval (model fitted to data from S1 to S7; see Table 1). The y-axis is log-transformed. (*B*) Proportion of exposed population with at least one parasite in the preintervention (1980–1989; S1 to S7) and post-intervention periods (1994–2017; S8 to S12 see Table 1), as inferred by the parasite transmission model. Solid lines are central estimates and shaded areas give the 90% credible interval (CrI). Where the solid lines cross the dashed line, this gives the age at which half of the exposed population become first infected; 2.2 y old (90% CrI 1.8 to 3.3 y) in the preintervention period and 8.5 y old (90% CrI 7.5 to 10.5 y) in the postintervention period. (*C*) Cumulative exposure to *O. viverrini* by age (Eq. **6**). The solid lines show the mean for the preintervention and postintervention periods, while the dot-dash lines show the cumulative worm burden for the upper 90% population percentile. Shaded areas give the 90% prediction interval. The y-axis is log-transformed. (*D*) Average lifespan of adult *O. viverrini* worms in human hosts. The posterior probability density is shown here for log(2)/σ; where σ is the spontaneous death rate of adult worms (Eq. **3**). The dashed line gives the posterior median (13 y). The filled area gives the 90% CrI (7 to 26 y).

The average age at which a person born between 1960 and 1989 was first infected with a single *O. viverrini* fluke is 2.2 y old (90% prediction interval [PI] 1.8 to 3.3 y). The average age of first infection for a person born after 1990 increases to 8.5 y (90% PI 7.5 to 10.5 y), although a smaller proportion of the population ultimately becomes exposed during their lifetime; 32% postintervention (90% CrI 26 to 39%) compared with 88% preintervention (90% CrI 83 to 91%). We define exposure to *O. viverrini* as the cumulative number of adult flukes acquired by a person over time, which is given by the area under the force of infection curve (Eq. **6**). In endemic regions of Thailand in the 1980s by ages 10, 20, and 30 y the median number of *O. viverrini* cumulative flukes was 12 (90% PI 8 to 16), 39 (90% PI 27 to 52), and 72 (90% PI 48 to 103) respectively (Fig. 2*C*). Following public health interventions in the 1990s (8), we estimate that the *O. viverrini* force of infection declined substantially, with an almost 40-fold reduction in the age-dependent transmission rate, resulting in the majority of individuals born after 1990 remaining uninfected at age 30 (median worm burden is zero). The distribution of *O. viverrini* parasites among human hosts shows high variation, as is characteristic for helminths (37), and for the most heavily infected decile in the preintervention period the cumulative burden is 691 worms by 30 y of age (90% PI 504 to 986) and 14 worms in the postintervention period (90% PI 10 to 21). These findings add weight to the reported declines in *O. viverrini* transmission in Thailand following control programs (7–9), though our analysis quantifies this effect in terms of worm burden, age of first exposure, and proportion of the population exposed.

### Lifespan of the Adult Worm.

The lifespan of helminths in human infections is considered to be in the order of one year to three decades, although this is rarely quantified (38). We estimate the lifespan of *O. viverrini* within our force of infection model as 12.9 y (90% CrI 7.4 to 25.7 y), which is defined as the average time for half of adult stage parasites to die within the human host in the absence of anthelmintic treatment (Eq. **3**; see *Materials and Methods*). Our posterior distribution for parasite mortality is long-tailed (Fig. 2*D*) with the top five percent of adult *O. viverrini* lifespans ≥25.7 y. This finding is consistent with a case report of the related liver fluke *C. sinensis*, which persisted in an emigrant for 26 y (39). We note that the posterior distribution of the worm mortality parameter is correlated with a force of infection parameter and therefore encourage the use of the interval for *O. viverrini* lifespan (7 to 26 y), rather than the point estimate, in future analyses.

### Latent and Induction Periods of Cholangiocarcinoma.

**Fig. 3.**
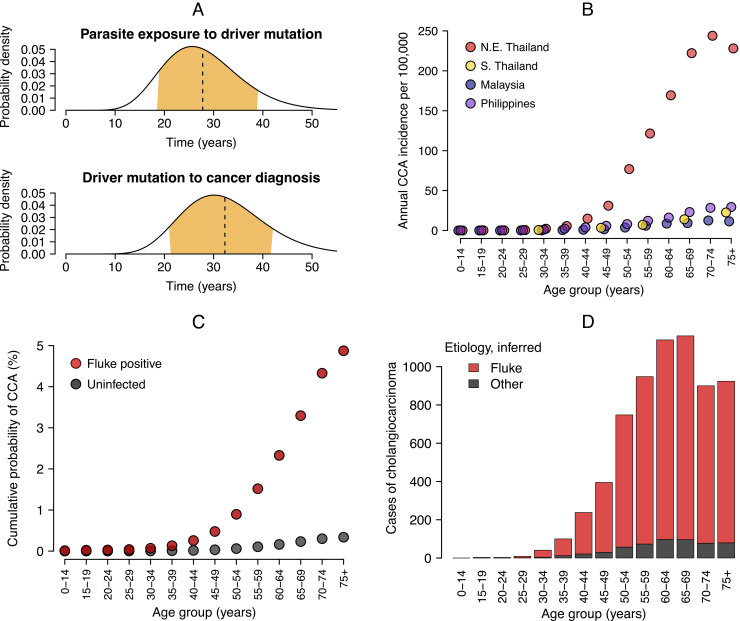
Incidence and age-distribution of cholangiocarcinoma (CCA). (*A*) Time-to-event distributions for the induction period (parasite exposure to driver mutation; median 28 y) and the latent period (driver mutation to cancer diagnosis; median 32 y). The plots show posterior probability distributions, where the dashed line gives the median and the orange area the 90% credible interval (CrI). (*B*) Annual incidence of cholangiocarcinoma per 100,000 people by age group for Northeast (N.E.) Thailand, where the carcinogenic liver fluke *Opisthorchis viverrini* is endemic, and Southern (S.) Thailand, Malaysia, and the Philippines, which are nonendemic for *O. viverrini*. (*C*) Cumulative lifetime probability of diagnosis with cholangiocarcinoma in Southeast Asia for i) a person infected with liver fluke (dark red) and ii) an uninfected person (gray). The model output is shown from a survival analysis (Eq. **16**; see *Materials and Methods*). Points give the posterior median probability within each age group. (*D*) The age distribution of 10,737 cases of cholangiocarcinoma from Northeast Thailand between 1985 and 2009 (40), colored by etiology (fluke or other) inferred from the survival model posterior parameters.

Bringing together the evidence presented thus far, we hypothesize that people born 1960–1989 in Northeast Thailand first became infected with liver fluke in early childhood and driver mutations for biliary cancer occurred around three decades later. We sought to validate our hypothesis using cancer registry data consisting of 10,737 cases of cholangiocarcinoma diagnosed in Northeast Thailand between 1985 and 2009 (40). The median age of diagnosis was 59 y between 1985 and 1997 and 63 y between 1997 and 2009. Using a time-to-event analysis, which accounts for interval censored data, we estimate the age of driver mutation and the subsequent age of cholangiocarcinoma diagnosis as sequential gamma distributions (*Materials and Methods*). The induction period (time from initial parasite infection to driver mutation) is estimated as 28 y (90% CrI 16 to 39 y) and the latent period (time from driver mutation to cancer diagnosis) is 32 y (90% CrI 21 to 44 y). These distributions are shown in Fig. 3*A*. The cancer incidence data therefore support our estimate of the time to first amplified mutation at around thirty years of age, given exposure by age two. The estimated latent period from registry data is longer than the 22 y indicated by our analysis of tumor genomes (time from first amplified driver mutation to biliary surgery), which likely reflects an older average age of diagnosis in the registry data compared to our much smaller sample of patients with sequenced biliary tumors. Previous research on the induction and latent periods for fluke-induced cholangiocarcinoma in humans is limited, though American veterans from the Vietnam War had an elevated incidence of biliary cancer five or more decades after potential exposure to *O. viverrini* between 1965 and 1971 (41). While the association between military action in Vietnam and fluke-induced cholangiocarcinoma is controversial (42), the reported time between parasite exposure and cancer is consistent with our findings.

### Lifetime Probability of Cholangiocarcinoma.

Finally, we estimate the lifetime probability of acquiring cholangiocarcinoma, and the increase in risk from parasite infection, by fitting a survival model (see *Materials and Methods* and Eqs. **16** and **17**) to age-incidence cancer registry data from Northeast Thailand between 1997 and 2009 (40). For comparison, we include cancer registry and mortality data from neighboring populations (Southern Thailand, Malaysia, and the Philippines) that are nonendemic for *O. viverrini* to estimate the baseline risk of cholangiocarcinoma in the absence of parasite infection. The underlying data are shown in Fig. 3*B* as the annual incidence of cholangiocarcinoma per 100,000 people by age. We obtain posterior parameters for age-variable intercepts ($\alpha_{a}$) and a coefficient (β), which gives the relative risk of diagnosis with cholangiocarcinoma given exposure to liver fluke, estimated as 2.73 on the logit scale; equating to an odds ratio of 15.3 (90% CrI 14.7 to 15.8). The model fit to cholangiocarcinoma incidence data is shown in *SI Appendix*, Fig. S5.

We use the posterior parameters to produce counterfactual scenarios for the lifetime probability of cholangiocarcinoma diagnosis given prior infection with liver fluke; see Fig. 3*C*. Our results show that by 75 y of age the cumulative probability of diagnosis with cholangiocarcinoma is 0.34% (90% CrI 0.33 to 0.35%) for a person in Southeast Asia uninfected with liver fluke and 4.9% (90% CrI 4.7 to 5.0%) for a person infected with liver fluke, which is fourteen-fold higher. Our estimate is similar to a previously reported value for Northeast Thailand using registry data from 1998 to 2002 (43), though we correct for the proportion of the population exposed to *O. viverrini* by age and to estimate the baseline risk of cholangiocarcinoma in the absence of parasite infection.

We calculate the number of excess cholangiocarcinoma cases in Northeast Thailand attributable to infection with liver fluke by assigning each age group a relative probability of i) baseline risk ($\alpha_{i}$) and ii) the elevated risk from liver fluke infection given the parasite exposure in that age group ($\alpha_{i}+\beta q_{i}$). Using these probabilities we estimate that 92% of cholangiocarcinoma cases between 1997 and 2009 are attributable to *O. viverrini* infection (Fig. 3*D*). A case–control study in Thailand previously estimated that 91% of cholangiocarcinoma cases in men and 80% in women were attributable to exogenous risk factors (12) including *O. viverrini* exposure along with betel nut chewing, which is known to be an oral carcinogen (44).

Given the availability of data, our estimate of cholangiocarcinoma risk is unstratified by host factors other than age, such as sex, nor is it provided at a more granular spatial scale. The probability of developing cholangiocarcinoma varies with the intensity of liver fluke infection (45) and, given improved longitudinal data, the lifetime probability of cancer should be stratified by the cumulative worm burden in future analyses.

## Discussion

This study uncovers crucial epidemiological processes on the pathway from parasite infection to malignancy (Table 2), and our objective is for these findings to inform control programs to reduce transmission of liver fluke and subsequent cases of cancer (16, 17). Given our findings that the host age at first fluke infection is before ten years of age and the first amplified driver mutations for biliary cancer occur three decades later, we recommend that interventions should target younger people in endemic regions, such as school-based deworming (46). There is limited evidence on the impact of anthelmintic treatment on preventing or reversing biliary damage in humans (47, 48), although current evidence suggests a beneficial effect of deworming even in the context of repeated reinfection. Given the dramatic fall in parasite transmission from 1990 in Thailand, we predict that the future incidence of cholangiocarcinoma will also decline, though the long temporal lags between initial parasite exposure, carcinogenesis, and cancer diagnosis (Figs. 2*B* and 3*C*) means that changes to *O. viverrini* transmission intensity will take many years to influence the incidence of cholangiocarcinoma at the population level (17). Randomized trials investigating the impact of deworming on the risk of biliary cancer could be considered unethical given the need to restrict anthelmintic treatment for controls (16). Dynamic simulations have a useful role to play, therefore, in estimating the magnitude of interventions over long periods (49). Our results also provide a platform for biomarker discovery by highlighting early driver genes and the long latent period for cholangiocarcinoma provides a window for early therapeutic interventions. Validating predictions of early cholangiocarcinoma driver genes is challenging in humans, although biobanking liver biopsies and bile samples provides an opportunity to prospectively characterize somatic evolution. Ideally, longitudinal studies would record detailed patient information on liver fluke infection and other carcinogenic exposures at multiple time points.

**Table 2.** Epidemiological parameters for the liver fluke *Opisthorchis viverrini* and the resulting biliary cancer (cholangiocarcinoma; CCA) estimated in this study using parasitological and cancer genomic data from Thailand

Parameter	Value^*^	Unit	Interval^†^	Data	Period
Age at first infection	2.2	Years	1.8 to 3.3 [PI]	S1 to S7	1960–1989
	8.5		7.5 to 10.5 [PI]	S8 to S12	1990–2017
Age at first CCA driver mutation^‡^	30	Years	20 to 43 [IQR]	CCA genomes	1950–2010
Driver mutation to CCA diagnosis	32	Years	21 to 44 [CrI]	Cancer registry	1998–2009
Population exposed to *O. viverrini*	88	%	83 to 91 [PI]	S1 to S7	1960–1989
	32		26 to 39 [PI]	S8 to S12	1990–2017
*O. viverrini* lifespan^§^	13	Years	7 to 26 [CrI]	S1 to S7	1960–1989
Parasite exposure at 30 y/old (median)^¶^	72	Flukes	43 to 110 [PI]	S1 to S7	1960–1989
	0		0 to 0 [PI]	S8 to S12	1990–2017
Parasite exposure at 30 y/old (high)^#^	691	Flukes	530 to 937 [PI]	S1 to S7	1960–1989
	10		8 to 14 [PI]	S8 to S12	1990–2017
Lifetime CCA risk |*O. viverrini* infection	4.9	%	4.7 to 5.0 [CrI]	Cancer registry	1998–2009

^*^Posterior median value.

^†^Types of uncertainty intervals are 90% prediction interval [PI], interquartile range [IQR], and 90% credible interval [CrI].

^‡^Age at first amplified driver gene, using C>T mutations at CpG sites under the assumption of a constant mutation rate (see *SI Appendix*, Fig. S1 for alternative scenarios).

^§^Lifespan of adult *O. viverrini* worms within the human host.

^¶^Cumulative parasite burden at 30 y of age given a median exposure (50th percentile).

^#^Cumulative parasite burden at 30 y of age given a high exposure (90th percentile).

The evolutionary cancer analysis in this study is limited in using single, rather than multiple, tumor and normal sequenced biopsies from each patient, which may result in subclones being under or over represented in the sample (50). The inferred age of amplified mutations is nevertheless similar for clonally and subclonally assigned variants (Fig. 1*C*), suggesting that our findings are robust. Capturing tumor heterogeneity and clonal inference in future studies would benefit from multisite sequencing or a single cell approach (51). Novel methods for assigning mutational signatures to SNVs to characterize multiple clock-like signatures and identify mutations specifically associated with parasite exposure would further enhance such studies (25, 52).

Relatively few studies have investigated the epidemiological relationship between infection and carcinogenesis, although compared against other pathogen-driven cancers our estimates of the induction and latent periods for fluke-induced cholangiocarcinoma appear longer. For human papillomavirus (HPV) the time from infection to high-grade cervical intraepithelial neoplasia (CIN2/3) has been estimated from registry data as 3 y, and the time from CIN2/3 to cancer 23.5 y (53). The time from hepatitis B infection to hepatocellular carcinoma is reported as 25 to 30 y in a review (54) though we are unaware of the supporting evidence. Mechanisms for a potentially longer tumorigenesis from *O. viverrini* infection, compared with other carcinogenic pathogens, have not been investigated. We note that the impact of fluke infection on biliary pathology is dose-dependent (45). There may also be evolutionary reasons for a slower accumulation of pathology from helminths, given their long lifespans (55).

We assume that excess cases of cholangiocarcinoma in Northeast Thailand (when compared against Southern Thailand, Malaysia, and the Philippines) are solely attributable to liver fluke exposure, though other host factors may be partly responsible. Betel nut chewing and the consumption of fermented foods containing nitrosamines have been implicated as additional risk factors in cross-sectional studies (12, 56). Betel nut chewing occurs in multiple Southeast Asian countries including the Philippines and Malaysia (44), which are used to estimate the baseline risk of cholangiocarcinoma (Fig. 3*B*). Therefore, our analysis controls for other carcinogenic exposures to the extent that they are practiced throughout Southeast Asia.

As the data used here are primarily from Northeast Thailand, our estimates of epidemiological parameters are most applicable to this region. Large-scale ultrasound screening programs for liver disease in Thailand have successfully diagnosed thousands of cholangiocarcinoma and precancerous cases since 2015 (57). However, enhanced screening will likely lead to short-term increases in the reported incidence of cholangiocarcinoma, further complicating our understanding of the relationship between parasite infection and cancer (17). Extending our analyses to Lao PDR and Cambodia, where the burden of parasitic disease is greater (7, 11) and the capacity for healthcare systems to diagnose and treat biliary cancer more limited (58), remains a priority for future research.

## Materials and Methods

### Cholangiocarcinoma Whole-Genome Sequences.

We accessed paired tumor-normal whole-genome sequences from 22 individuals in Northeast Thailand with previous exposure to liver fluke infection. Tumor tissue was obtained from patients during surgical resection of the biliary tract at Srinagarind Hospital in Khon Kaen, Thailand, and sequencing was performed on a single core sample per-patient (20). Normal somatic genomes were obtained from patient blood samples. Tumors are classified according to their anatomical location on the biliary tree; namely intrahepatic cholangiocarcinoma (within the hepatic ducts), perihilar cholangiocarcinoma (between the second-order bile ducts and the cystic duct insertion), or distal cholangiocarcinoma (below the cystic duct). Here, our samples consist of eight perihilar and 14 intrahepatic tumors. The age at surgery ranged from 37 to 79 y (median 57 y) and 11/22 (50%) of patients were female.

### Cancer Genomics.

We trimmed adaptor sequences from 150bp Illumina paired-end reads and mapped the reads against the human reference (GRCh37) using bwa mem v.0.7.17 with default parameters (59). The resulting BAM files were then sorted and indexed with samtools v.1.9. Duplicates were marked and removed using GATK v.4.1.4.1 (60) and base quality scores recalibrated for tumor sequences with ICGC PCAWG consensus calls for somatic SNVs and indels (61). To examine the depth of mapped reads, we took the output from samtools depth -a (including zeroes) and binned the mean depth within 1Mb segments. Sequencing coverage ranged from 35 to 73× per sample (median 57×) for normal genomes and 45 to 72× (median 54×) for tumor genomes.

We called SNVs and indels using GATK Mutect2 with a panel of normals provided by the Broad Institute and additional filters to remove secondary and supplementary reads. Before filtering, the number of SNVs ranged from 109,339 to 292,886 per sample (median 131,057). We calculated the fraction of normal reads with tumor contamination using the GATK tool CalculateContamination in combination with 4.7 million common germline alleles (MAF 0.01 to 0.20) derived from Asian populations in Singapore (62). This revealed that contamination in normal samples was low with <0.7% of reads coming from cross-sample contamination. Using the contamination data we filtered the variant calls, leaving 2,349 to 27,821 (median 10,360) SNVs and 268 to 14,230 (median 1,382) indels per tumor. The number of SNVs called are consistent with other biliary cancers (61). See *SI Appendix*, Table S1 for a summary of tumor genomes and metadata.

### Subclonal Reconstruction.

We estimate tumor copy number using the Battenberg algorithm (63), with reference data from the 1000 Genomes Project. The estimated fraction of tumor cells (rather than normal tissue) in our cholangiocarcinoma genomes, also known as tumor purity or cellularity, varied substantially between samples (range 10 to 90%; median 60%). The overall ploidy per tumor ranged from 1.2 to 3.7 (median 2.0). We then phased the somatic variants and assigned them to subclonal lineages (24) using dpclust3p and dpclust (64), implemented in R v.4.3.2. The number of clonal and subclonal lineages, to which variants were assigned, varied from 2 to 5 per tumor (median 3).

### Timing of Driver Mutations.

We applied algorithms to estimate the chronological timing of driver mutations in amplified regions; MutationTimeR (19) and AmplificationTimeR (23), which were implemented in R v.4.3.3. The MutationTimeR algorithm uses a panel of 371 known driver mutations identified by the PCAWG consortium (61). We used the temporal correlation of copy number gains from MutationTimeR to identify whether tumors had undergone whole-genome duplication (19). For the AmplificationTimeR analysis, we focused on timing the amplification of 43 driver mutations that have been identified as important in early-stage cholangiocarcinoma (21, 22) or previously detected in these tumors (20). Estimates for the chronological time of amplifications were calibrated using C>T mutations at CpG sites (CpG>TpG), which have been established to have clock-like properties and the mutation burden correlates with age (25). The age at driver gene amplification was calculated as the product of the chronological time estimates from AmplificationTimeR (23) in the interval [0, 1] and the patient’s age at surgery (20) under the assumption of a constant CpG>TpG mutation rate. For scenarios where the CpG>TpG rate accelerates with age in tumor cells see *SI Appendix*, Fig. S1.

### Earliest and Latest Amplified Genes.

We used a generalized linear model to determine which genes were amplified disproportionately early or late during tumorigenesis. We modeled the chronological time estimates for gene g$\left(y_{t,g}\right)$, estimated by AmplificationTimeR (23) with a minimum of 10 CpG>TpG mutations, which fall in the interval [0,1] using a beta distribution parameterized by a mean $\mu_{g}$ and precision κ

[1] $$\begin{array}{c} y_{t,g}∼Beta\_Proportion\left(\mu_{g},\kappa \right), \end{array}$$

where the mean is a transformed linear function of a gene-specific intercept ($\alpha_{g}$) plus covariates (x) and slopes (β). The three binary explanatory variables are the sex of the patient, whether the tumor is intrahepatic or perihilar, and whether the amplification is clonal or subclonal

[2] $$\begin{array}{c} \mu_{g}=logit^{-1}(\alpha_{g}+\beta_{1}x_{1}+\beta_{2}x_{2}+\beta_{3}x_{3}). \end{array}$$

The model was fitted in a Bayesian framework using the stan language v.2.34.1 (65) implemented with cmdstanr v.0.7.1 in R v.4.3.3; see details on parameter estimation below.

### Liver Fluke Surveys.

We obtained data from epidemiological surveys on the liver fluke *O. viverrini* in Thailand between 1980and 2017. The parasitological observations in these surveys consist of i) adult worms obtained from liver dissection at autopsy, ii) adult worms recovered through expulsion following anthelminthic treatment, or iii) parasite egg counts obtained by fecal examination; see Table 1. We contacted the study authors to obtain parasitological data by host age. Where these were unavailable, we simulated individual-level data from summary tables which contained the sample size, mean, and variance for parasitological observations (worm burdens or fecal egg counts) aggregated by age group using a Pearson type I distribution. Individual-level data are shown in *SI Appendix*, Fig. S2, in addition to prevalence by age (*SI Appendix*, Figs. S3 and S4).

### Parasite Transmission Model.

We developed a mechanistic model that incorporates key aspects of parasite ecology (35, 66) fitted to individual-level data on adult worm burdens or fecal egg counts (data summarized in Table 1). Our process model characterizes the mean worm burden at the population level (M) by host years of age (a) as an immigration-death process

[3] $$\begin{array}{c} \frac{\mathrm{dM}}{\mathrm{da}}=\lambda \left(a\right)-M\sigma , \end{array}$$

where σ gives the spontaneous death rate of adult worms in the absence of anthelmintic treatment. The expected lifespan of adult *O. viverrini* parasites is therefore given as the time taken for half of adult worms to die in the absence of anthelmintic treatment; log(2)/σ. As there is evidence for age-dependent reinfection rates (67), we model the *O. viverrini* force of infection as a function of host age

[4] $$\begin{array}{c} \lambda \left(a\right)=\eta ae^{-\beta a}. \end{array}$$

The dynamic model (Eqs. **3** and **4**) has the following analytical solution for worm burden by age a,

[5] $$\begin{array}{c} M\left(a\right)=\frac{\eta e^{-a\sigma }}{{\left(\beta -\sigma \right)}^{2}}(1+e^{a(\sigma -\beta )}\left(a\left(\sigma -\beta \right)-1\right)). \end{array}$$

The cumulative parasite exposure (δ) by age a is given by the definite integral of the force of infection (Eq. **4**)

[6] $$\begin{array}{c} \delta \left(a\right)=\int_{0}^{a}\eta ae^{-\beta a}\;da. \end{array}$$

### Observation Model for Survey Data.

The true number of *O. viverrini* adult worms per individual i of age group a ($x_{i,a}$) follows the negative binomial distribution (NB), which takes the form of a gamma-Poisson mixture model parameterized with a mean worm burden M(a) (Eqs. **3** and **5**) and age-dependent dispersion $k_{a}$ (36). Values of $k_{a}$ are estimated for each age-group and are themselves normally distributed with an overall mean of $\mu_{k}$ and a SD of $\sigma_{k}$. During autopsy surveys, adult *O. viverrini* were carefully removed from cross-sections of liver and worm recovery is likely close to 100%, therefore we consider that the true worm count for each individual is equal to the recovered worms in autopsy studies ($x_{i,a}\equiv w_{i,a}\;|\;r=1$),

[7] $$\begin{array}{cc} Pr(X_{i,a} & =x_{i,a}\;|\;M\left(a\right),k_{a})\\ & =NB(x_{i,a}\;|\;M\left(a\right),k_{a}). \end{array}$$

In surveys where *O. viverrini* flukes were obtained by expulsion, participants were treated with the anthelmintic praziquantel followed by a saline purgative, which is known to result in imperfect subsequent recovery of from feces (68). Therefore, we allow the true worm burden to be greater than, or equal to, the observed number of worms for individuals in expulsion studies ($x_{i,a}\geq w_{i,a}$) and the probability of observing $w_{i}$ worms given a true count of $x_{i}$ is a binomial sampling process with probability of worm recovery r=0.44 (36),

[8] $$\begin{array}{cc} Pr(W_{i,a} & =w_{i,a}|M\left(a\right),k_{a},r)\\ & =\sum_{x=w_{i,a}}^{\infty }Binom\left(w_{i,a}\;|\;x,r\right)\cdot NB(x\;|\;M\left(a\right),k_{a}), \end{array}$$

where *Binom* refers to a standard binomial probability distribution. In surveys where the outcome variables are eggs per gram of stool (y), if at least one egg is observed for individual i of age group a ($y_{i,a}\geq 1$), we relate this to the expected egg count for that individual using a negative binomial error distribution, where the mean is given as a density-dependent function of the true worm burden; $\pi \left(x\right)={\left(\Lambda x\right)}^{\gamma }$ and the dispersion is given by parameter h, which has previously been estimated for *O. viverrini* as 0.4 (36),

[9] $$\begin{array}{cc} Pr(Y_{i,a} & =y_{i,a}\;|\;M\left(a\right),k_{a},\Lambda ,\gamma ,h)\\ & =\sum_{x=1}^{\infty }NB(y_{i,a}\;|\;\pi \left(x\right),h)\cdot NB(x\;|\;M\left(a\right),k_{a}). \end{array}$$

Where zero eggs are observed ($y_{i,a}=0$), we consider the individual diagnostic sensitivity as a saturating function of the worm burden, se(x)=x/(x+b), where the parameter b has been previously estimated for *O. viverrini* as 1.7 (36),

[10] $$\begin{array}{cc} Pr(Y_{i,a} & =0\;|\;M\left(a\right),k_{a},b)\\ & =\sum_{x=0}^{\infty }(1-\frac{x}{x+b})\cdot NB(x\;|\;M\left(a\right),k_{a}). \end{array}$$

The population level sensitivity (S) for fecal egg diagnostics is a function of the worm burden distribution at the population level (36),

[11] $$\begin{array}{c} S\left(M,k\right)=\sum_{x=1}^{\infty }se\left(x\right)\cdot \left(\begin{array}{c} x+k-1\\ x \end{array}\right)\frac{{\left(\frac{k}{M+k}\right)}^{k}{\left(\frac{M}{M+k}\right)}^{x}}{p(M,k)}, \end{array}$$

where p(M,k) indicates the true prevalence and is given by

[12] $$\begin{array}{c} p\left(M,k\right)=1-{\left(\frac{k}{M+k}\right)}^{k}. \end{array}$$

Given an assumed diagnostic specificity of one, the observed prevalence $p^{\prime }$ for for age group a is therefore related to the true prevalence with the following expression,

[13] $$\begin{array}{c} p^{\prime }=p\left(M,k\right)\cdot S\left(M,k\right)+(1-p\left(M,k\right))\cdot \left(1-sp\right), \end{array}$$

where *sp* gives the fecal egg diagnostic specificity, which is assumed here to be 1. For surveys where only the prevalence is given by fecal egg diagnostic, we represent this as individual-level positive or negative outcomes; $z_{i,a}\in \left\{0,1\right\}$. The probability for the binary diagnostic observations is therefore given by a Bernoulli distribution

[14] $$\begin{array}{cc} Pr(Z_{i,a} & =z_{i,a}\;\left|\;M\left(a\right),k_{a},b\right)\\ & =\left\{\begin{array}{cc} p_{a}^{\prime } & \text{if}\;z_{i,a}=1\\ 1-p_{a}^{\prime } & \text{if}\;z_{i,a}=0. \end{array}\right. \end{array}$$

### Epidemiological Parameter Estimation.

Model fitting was performed in a Bayesian framework using the stan language v.2.34.1 (65) implemented with cmdstanr v.0.7.1 (69) in R v.4.3.3. Parameters were assigned weakly or moderately informative prior distributions based on the results from a previous analysis (36) for the preintervention data (S1 to S7; see Table 1). For the postintervention analysis (S8 to S12), several model parameter values were taken directly from the preintervention posterior distributions; including the parameters relating worm burdens to egg counts (Λ, γ) and the worm mortality rate (σ), which was taken as the highest percentile of the posterior estimate (σ=0.116), corresponding to a mean worm lifespan of 6 y, to account for higher parasite mortality resulting from periodic anthelmintic treatment after 1990. Each model was run with four parallel chains with a burn-in of 1,700 iterations per chain and a total of 1,000 iterations. The Gelman–Rubin diagnostic $\hat{r}\leq 1.01$ and effective sample size >500 were used to diagnose successful Markov chain convergence. Results are presented as credible intervals (CrI) of parameter posterior distribution or prediction intervals (PI) from simulations. All prior and posterior parameters from the parasite transmission model are given in *SI Appendix*, Table S2.

### Induction and Latent Period.

We performed a time-to-event analysis to validate the induction and latent periods estimated in the evolutionary cancer analysis using cancer registry data. We obtained the age distribution of 10,737 cholangiocarcinoma cases diagnosed at Srinagarind Hospital between 1985 and 2009 (40) that were grouped into age classes. We consider that for each cancer case at age a there is an unknown age at which a driver mutation was obtained m, where m<a. Values of m are drawn from a gamma distribution with mean $\mu_{\mathrm{mut}}$ and shape parameter $\alpha_{\mathrm{mut}}$. Following the mutation at age m there is a latent period before cancer diagnosis at age a, this latent period is also gamma distributed with mean $\mu_{\mathrm{cca}}$ and shape $\alpha_{\mathrm{cca}}$. The probability of developing cholangiocarcinoma at age a, $\phi_{a}$ is therefore

[15] $$\begin{array}{cc} \phi_{a} & =\sum_{m=1}^{a-1}Gamma(m\;|\;\mu_{\mathrm{mut}},\alpha_{\mathrm{mut}})\\ & \cdot \;Gamma(a-m\;|\;\mu_{\mathrm{cca}},\alpha_{\mathrm{cca}}). \end{array}$$

### Probability of Acquiring Cholangiocarcinoma.

We adopt a binomial regression framework with the probability of contracting cholangiocarcinoma in age group i, population j given by

[16] $$\begin{array}{c} \theta_{i,j}=logit^{-1}(\alpha_{i}+\beta q_{i,j}), \end{array}$$

where α is an intercept which varies by age group i and β is a coefficient multiplied by the proportion of the population exposed to liver fluke q. For the population in Northeast Thailand, $q_{i,j}$ is taken as the preintervention prevalence for age groups ≥20 y old and the postintervention prevalence for age groups <20 y old. In nonfluke populations (Southern Thailand, Malaysia, and the Philippines) $q_{i,j}=0$. Counts of cholangiocarcinoma cases $c_{i,j}$ are reported by age group i with an underlying population size of $N_{i,j}$. The likelihood is therefore given by

[17] $$\begin{array}{c} Pr(C_{i,j}=c_{i,j}\left|N_{i,j},\theta_{i,j})=Binom(c_{i,j}\;\right|\;N_{i,j},\theta_{i,j}). \end{array}$$

To calculate the lifetime risk of cholangiocarcinoma under the counterfactual scenarios of either infection with liver fluke (q=1) or uninfected (q=0), we obtain the adjusted unconditional probability of cholangiocarcinoma by summing the probabilities in each five year age interval i∈{1,2,...,m}

[18] $$\begin{array}{c} \sum_{i=1}^{m}(1-{\left(1-\theta_{i,j}\right)}^{5})\cdot \rho_{i,j}, \end{array}$$

where $\rho_{i,j}$ is the probability of survival to age group i given by demographic life tables for Thailand and Malaysia (70). For the fluke-endemic population in the survival analysis, we used counts of cholangiocarcinoma cases from the cancer registry at Srinagarind Hospital in Khon Kaen (40) between 1998 and 2009 along with corresponding demographic data (N) in the same period (71). Cholangiocarcinoma case data from Southern Thailand (Songkla province) between 2004 and 2013 was used to calculate the baseline risk (72), along with cancer and demographic data for Malaysia (2007–2009) and the Philippines (2008) obtained from the World Health Organization Mortality Database using version 10 ICD codes C22 (malignant neoplasm of liver and intrahepatic bile ducts) and C24 (malignant neoplasm of other and unspecified parts of biliary tract) (73). We assumed that half the biliary cancers in Malaysia and the Philippines were attributable to cholangiocarcinoma (14) and that, given the very low survival rate, mortality is a valid approximation for incidence (15).

### Ethical Statement.

As an analysis of previously published and anonymous human data, this study met the criteria for exemption from ethical review at the Universities of Oxford and Glasgow.

## Supplementary Material

Appendix 01 (PDF)

## Data Availability

This study uses previously published datasets. Code to reproduce the epidemiological analysis is available at https://github.com/tc13/ov-cca-models/ (74). Cholangiocarcinoma whole-genomes were accessed from the European Genome–Phenome archive under accessions EGAD00001001988 (75) and EGAD00001003834 (76).

## Footnotes

^*^https://publications.iarc.who.int/79.

^†^https://publications.iarc.who.int/119.
