# Supplementary material for: Natural history of liver fluke infection underpins epidemiological patterns of biliary cancer
Source: Proc Natl Acad Sci U S A. 2025 Oct 10;122(41):e2423536122. doi: 10.1073/pnas.2423536122 (PMC12541340; doi:10.1073/pnas.2423536122)
Supplement: Supplementary file 1 — Appendix 01 (PDF) [file pnas.2423536122.sapp.pdf]

Supporting Information for

Natural history of liver fluke infection underpins patterns of biliary cancer in an endemic population

Thomas Crellen, Francesca Vita, Chiara Braconi, Paiboon Sithithaworn, and T. Déirdre Hollingsworth

Corresponding author: Thomas Crellen.
E-mail: thomas.crellen@ndm.ox.ac.uk

This PDF file includes:

Figs. S1 to S5
Tables S1 to S2
SI References

Patient ID	Sex	Age[1] (years)	Anatomical subtype	Stage	Normal depth	Tumor depth	SNVs	Purity (%)	Ploidy	Clonal clusters
CCA_TH_1	F	49	Intrahepatic	III	66	64	5,209	77	2.0	3
CCA_TH_2	M	46	Perihilar	IIIA	43	45	4,362	59	1.5	3
CCA_TH_3	F	76	Perihilar	IIIA	58	45	15,064	63	2.7	4
CCA_TH_4	M	53	Perihilar	IVB	55	53	2,966	10	2.0	2
CCA_TH_5	M	60	Perihilar	IVA	64	62	12,744	42	3.7	2
CCA_TH_6	F	51	Intrahepatic	IVA	53	53	2,349	13	1.7	3
CCA_TH_7	M	61	Perihilar	IIIB	54	59	12,594	56	2.8	2
CCA_TH_8	F	53	Intrahepatic	III	64	54	5,828	45	1.9	4
CCA_TH_9	M	48	Intrahepatic	III	72	72	12,124	79	1.7	4
CCA_TH_10	F	69	Intrahepatic	IVA	61	53	13,432	63	2.5	2
CCA_TH_11	F	79	Intrahepatic	IVA	45	48	5,359	65	1.2	3
CCA_TH_12	M	52	Perihilar	IIIB	54	53	3,875	13	1.6	3
CCA_TH_13	M	57	Perihilar	IIIA	52	45	12,839	90	2.2	3
CCA_TH_14	F	64	Intrahepatic	III	63	62	10,527	73	2.9	2
CCA_TH_15	M	37	Perihilar	IIIA	60	63	13,290	34	3.2	2
CCA_TH_16	F	61	Intrahepatic	III	35	48	27,821	49	1.9	4
CCA_TH_17	M	66	Intrahepatic	III	69	61	7,260	47	1.5	5
CCA_TH_18	M	56	Intrahepatic	IVA	73	65	20,293	66	2.8	3
CCA_TH_19	F	65	Intrahepatic	III	71	68	8,341	61	2.8	3
CCA_TH_20	M	63	Intrahepatic	IVA	54	55	10,194	60	1.7	3
CCA_TH_21	F	40	Intrahepatic	I	54	55	5,115	79	1.9	4
CCA_TH_22	F	56	Intrahepatic	II	53	51	13,237	43	2.3	4

Table S1. Cholangiocarcinoma patients from Northeast Thailand with whole-genome sequenced normal and tumor samples. Metadata in columns 1–5 and cancer genomic parameters in columns 6–13. [1]Age at surgery. Metadata and raw Illumina 150bp paired reads from (1).

Parameter	Description	Value[1]	Unit	Interval[2]	Prior	Data	Period
η	Force of infection parameter	0.96	–	0.81–1.17	gamma(2, 1)	S1–S7	1960–1989
		0.013		0.0082–0.022	gamma(2, 1)	S8–S12	1990–2017
β	Force of infection parameter	0.030	–	0.019–0.044	gamma(1, 20)	S1–S7	1960–1989
		0.022		0.0089–0.036	gamma(1, 20)	S8–S12	1990–2017
σ	Death rate of adult worms	0.054	–	0.027–0.094	gamma(4, 40)	S1–S7	1960–1989
		0.12		–	Assumed	This study	1990–2017
k	Dispersion of adult worms						
	Age 0–4 yrs	0.27	–	0.17–0.40	normal(μ_k, σ_k)	S1–S7	1960–1989
	Age 5–9 yrs	0.23	–	0.20–0.26	normal(μ_k, σ_k)	S1–S7	1960–1989
	Age 10–19 yrs	0.43	–	0.39–0.47	normal(μ_k, σ_k)	S1–S7	1960–1989
	Age 20–29 yrs	0.36	–	0.32–0.40	normal(μ_k, σ_k)	S1–S7	1960–1989
	Age 30–39 yrs	0.36	–	0.33–0.41	normal(μ_k, σ_k)	S1–S7	1960–1989
	Age 40–49 yrs	0.35	–	0.31–0.39	normal(μ_k, σ_k)	S1–S7	1960–1989
	Age 50–59 yrs	0.37	–	0.32–0.42	normal(μ_k, σ_k)	S1–S7	1960–1989
	Age 60+ yrs	0.43	–	0.37–0.51	normal(μ_k, σ_k)	S1–S7	1960–1989
μ_k	Hyperparameter for k (mean)	0.35	–	0.28–0.41	gamma(3.3, 10)	S1–S7	1960–1989
		0.11		0.077–0.16	gamma(3.3, 10)	S8–S12	1990–2017
σ_k	Hyperparameter for k (variance)	0.086	–	0.049–0.16	exponential(2)	S1–S7	1960–1989
		0.027		0.0091–0.073	exponential(2)	S8–S12	1990–2017
Λ	Fecundity (eggs per worm)	79.6	–	75.2–84.1	gamma(710, 10)	S1–S7	1960–1989
		79.6		–	Assumed	This study	1990–2017
γ	Fecundity (density dependence)	0.89	–	0.86–0.92	beta(15, 2)	S1–S7	1960–1989
		0.89		–	Assumed	This study	1990–2017
h	Egg count dispersion	0.40	–	–	Assumed	(2)	1960–2017
r	Worm recovery from expulsion	0.44	–	–	Assumed	(2)	1960–2017
b	Fecal egg sensitivity parameter	1.70	–	–	Assumed	(2)	1960–2017
sp	Fecal egg specificity	1.0	–	–	Assumed	(3)	1960–2017

Table S2. Parameters estimated or assumed in the parasite transmission model. [1]Posterior median. [2]90% credible interval.

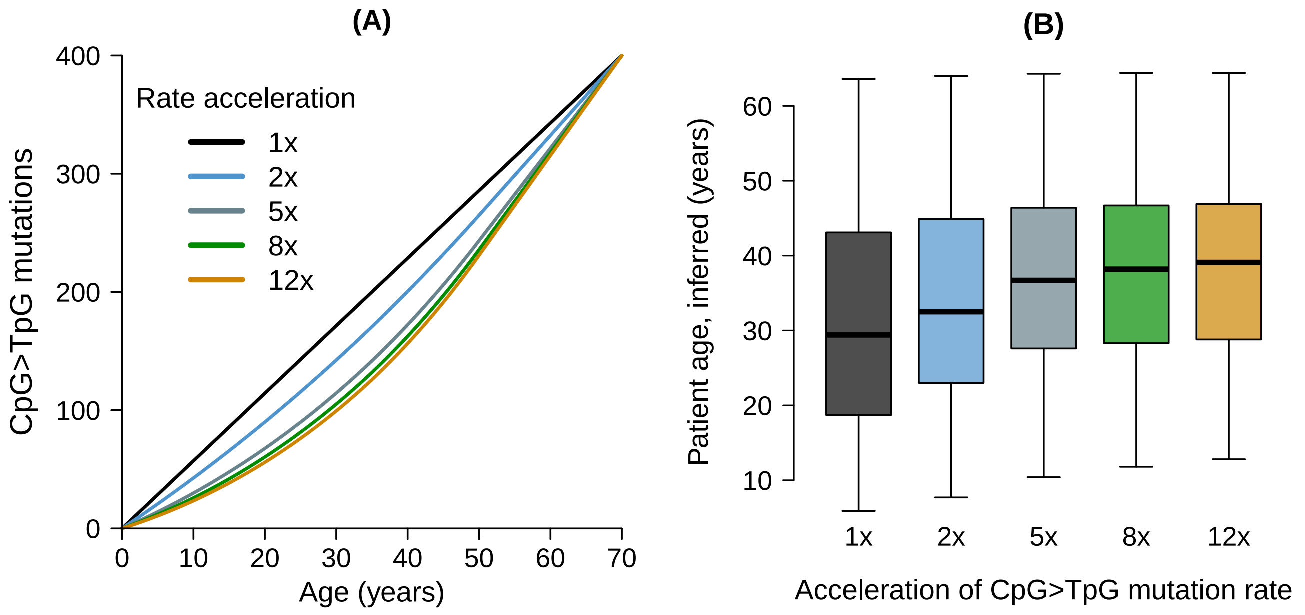

Fig. S1. Acceleration of the mutation rate used for timing somatic chromosomal amplifications. The cumulative trajectory for C>T mutations at CpG sites (CpG>TpG) is shown in panel A under scenarios where the mutation rate is either constant with age (1x) or accelerates by a factor of 2, 5, 8 or 12. The total number of observed somatic mutations is fixed at the age that the tumor biopsy is taken (in this example 400 mutations at 70 years of age). In scenarios where the rate accelerates with age, therefore, a greater proportion of the somatic mutational load occurs later in a patient's lifetime. The boxplots in panel B give the distribution of the age of first clonally amplified driver mutations in 17 fluke-induced cholangiocarcinoma tumors analyzed in this study under five scenarios for the CpG>TpG mutation rate. The inferred median ages of first clonal amplified driver genes are 30, 33, 36, 38 and 39 years respectively, given the different rates.

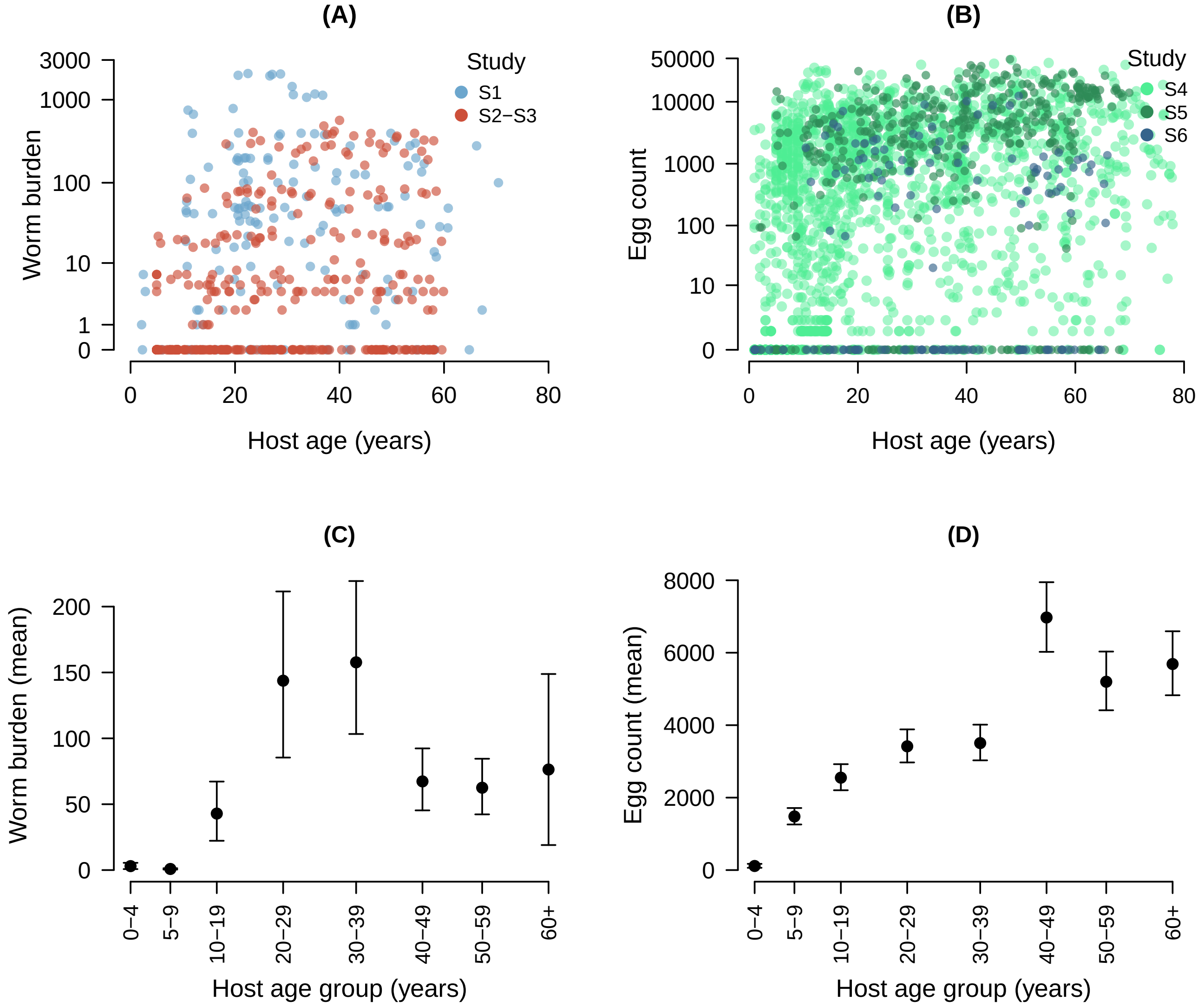

Fig. S2. Pre–intervention parasitological data showing individual *Opisthorchis viverrini* worm burdens (A) and fecal egg counts (B) by host age, where the points are colored by study (see data Table in main text). The aggregated mean worm burdens by age group are shown in panel C and the mean egg counts in panel D, where the uncertainty bars give the 90% confidence intervals by bootstrapping.

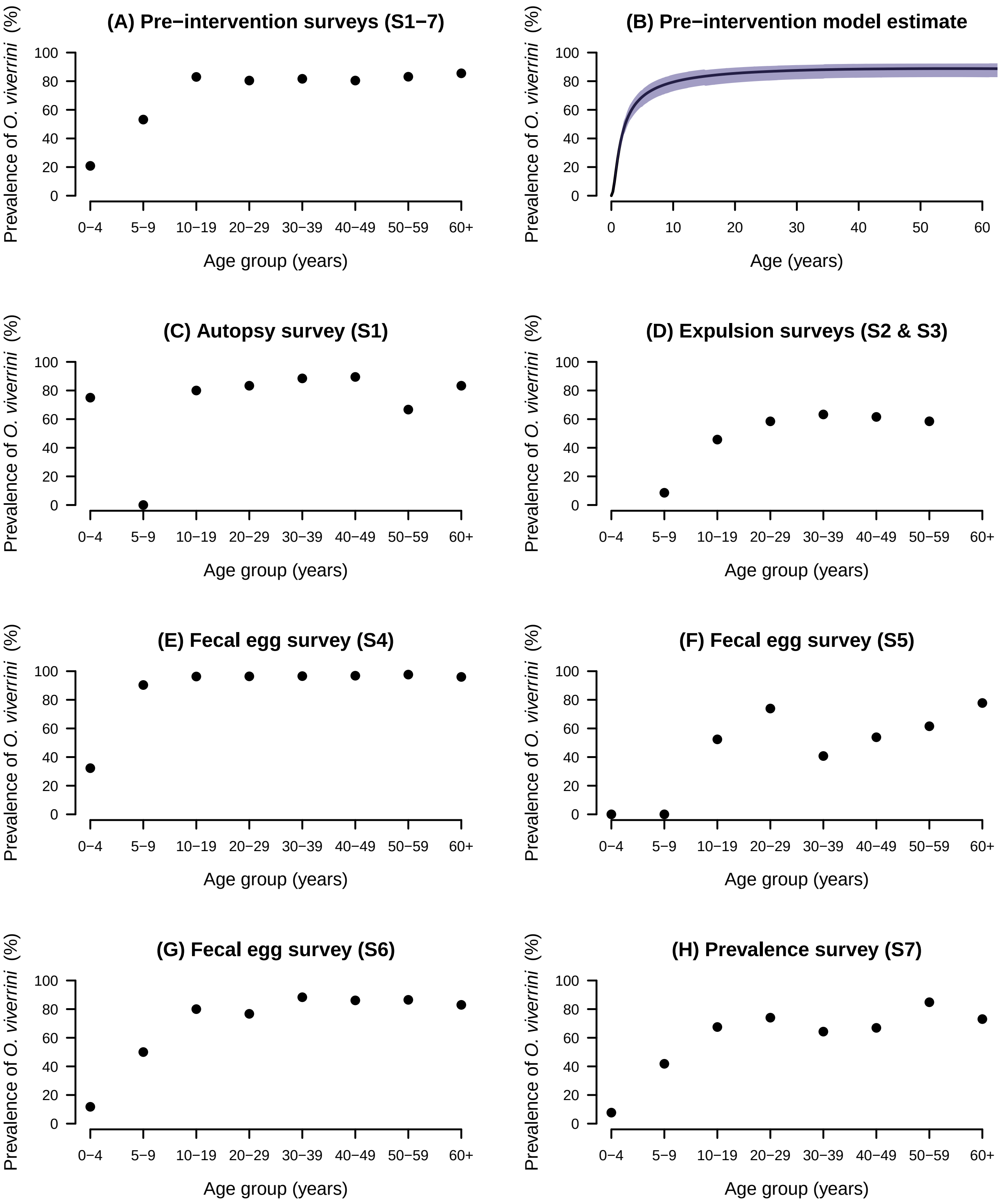

Fig. S3. Pre–intervention parasitological data showing the observed percentage of *Opisthorchis viverrini* cases by age group from surveys in Northeast Thailand between 1980–1989. Panel A shows the proportion positive by age for all surveys. The corresponding estimate of *O. viverrini* prevalence by age from the epidemiological model is shown in panel B, where the solid line gives the posterior median and the blue shaded area gives the 95% highest posterior density interval. Data from individual surveys are shown in panels C–H; see Table 1 in the main text for details.

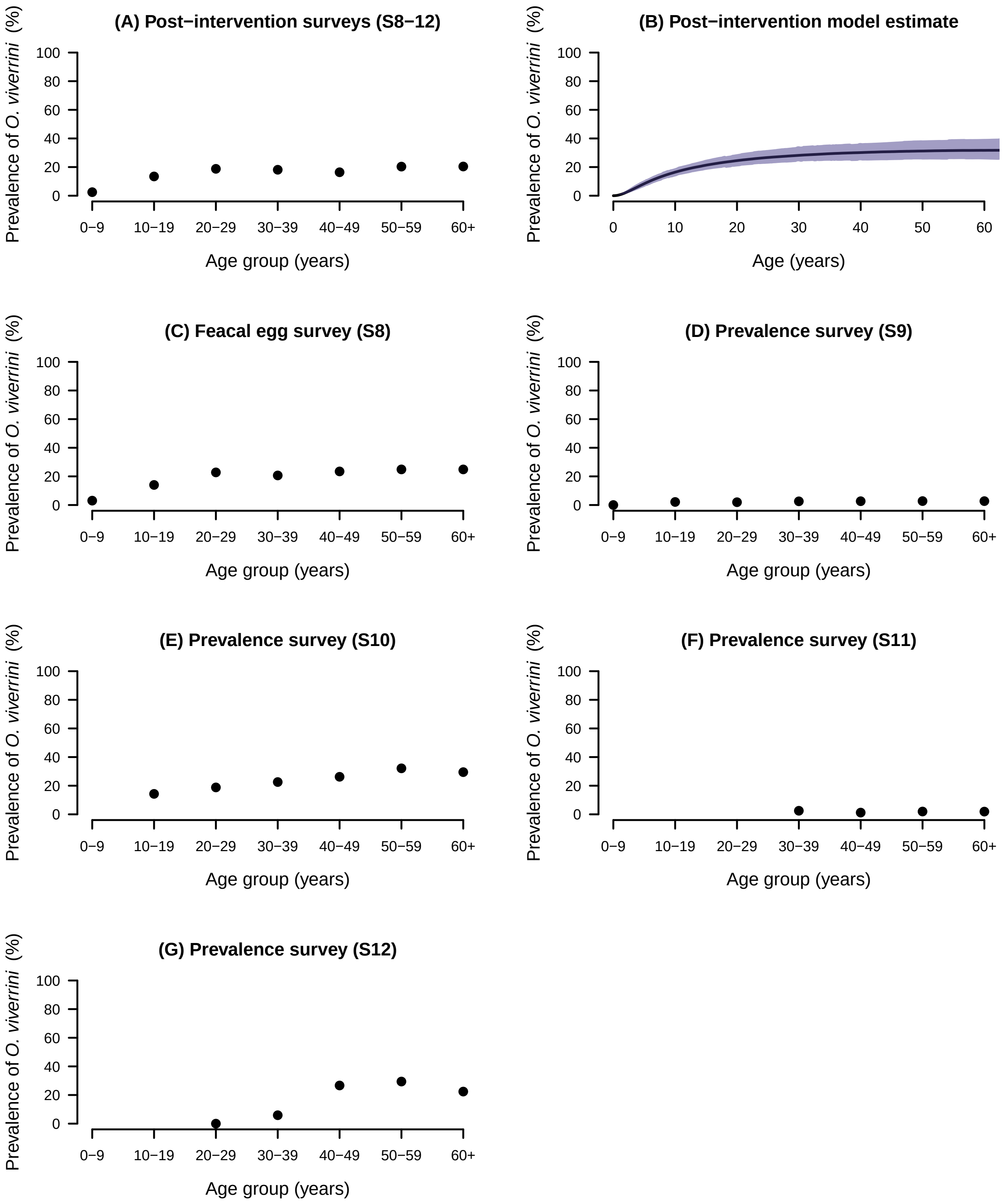

Fig. S4. Post–intervention parasitological data showing the observed percentage of *Opisthorchis viverrini* cases by age group from surveys in Northeast Thailand between 1994–2017. Panel A shows the proportion positive by age for all surveys. The corresponding estimate of *O. viverrini* prevalence by age from the epidemiological model is shown in panel B, where the solid line gives the posterior median and the blue shaded area gives the 95% highest posterior density interval. Data from individual surveys are shown in panels C–G; see Table 1 in the main text for details.

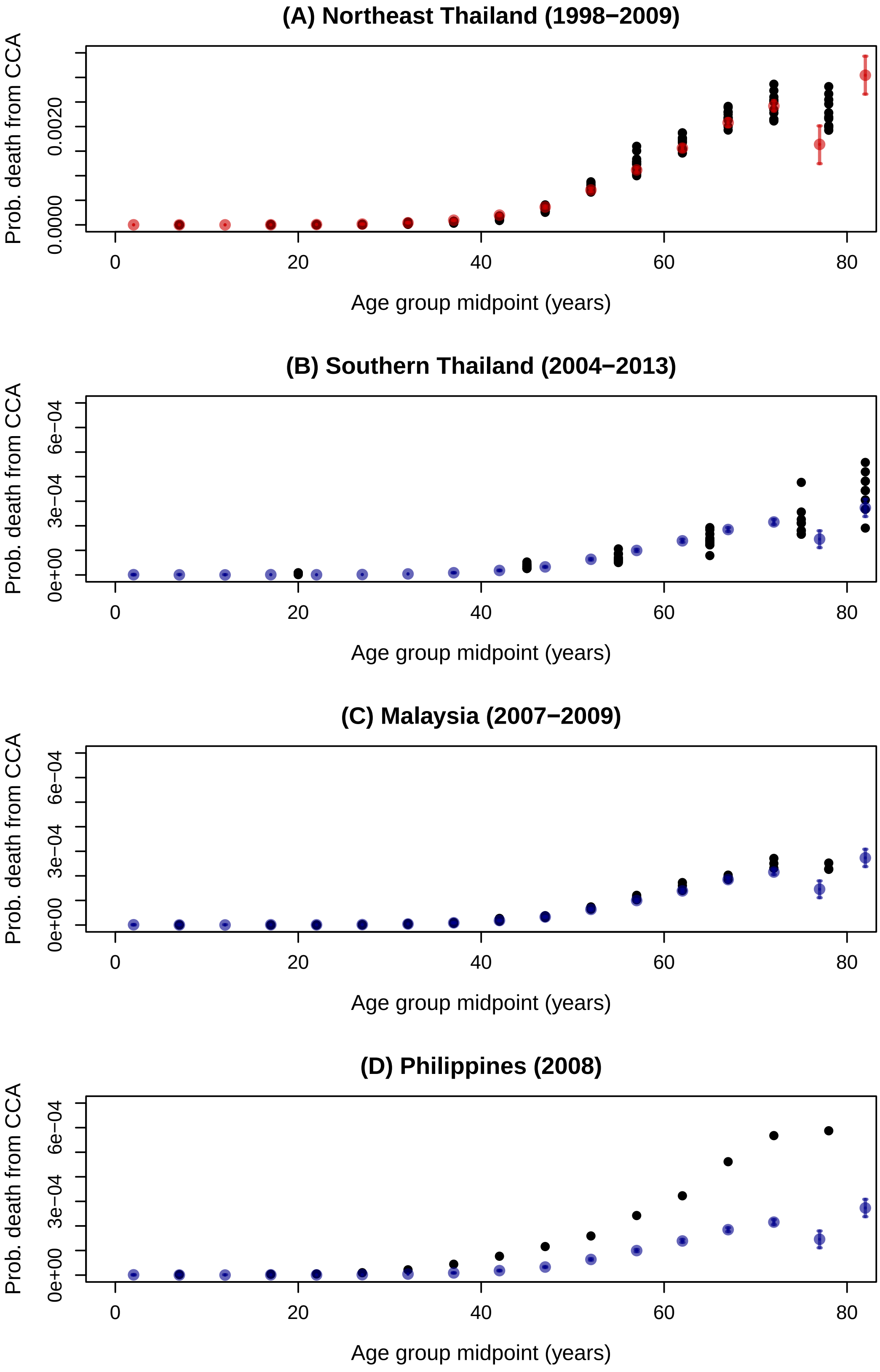

Fig. S5. Probability of diagnosis with cholangiocarcinoma (CCA) by age in Southeast Asia, conditional on survival to that age. Black points show data within intervals (4–6), coloured points show the survival model posterior median for age groups of five years, with a 90% credible interval. Red points show the estimates for Northeast Thailand, which is endemic for the liver fluke *Opisthorchis viverrini*, and blue points show the (baseline) model fit to three populations where *O. viverrini* is non–endemic.

References

1. A Jusakul, et al., Whole-genome and epigenomic landscapes of etiologically distinct subtypes of cholangiocarcinoma. *Cancer discovery* **7**, 1116–1135 (2017).
2. T Crellen, et al., Diagnosis of helminths depends on worm fecundity and the distribution of parasites within hosts. *Proc. Royal Soc. B* **290**, 20222204 (2023).
3. C Worasith, et al., Comparison of a urine antigen assay and multiple examinations with the formalin-ethyl acetate concentration technique for diagnosis of opisthorchiasis. *The Am. J. Trop. Medicine Hyg.* **109**, 159 (2023).
4. S Kamsa-ard, et al., Trends in liver cancer incidence between 1985 and 2009, Khon Kaen, Thailand: cholangiocarcinoma. *Asian Pac J Cancer Prev* **12**, 2209–2213 (2011).
5. S Yeesoonsang, et al., Trends in Incidence of Two Major Subtypes of Liver and Bile Duct Cancer: Hepatocellular Carcinoma and Cholangiocarcinoma in Songkhla, Southern Thailand, 1989–2030. *J. Cancer Epidemiol.* **2018**, 8267059 (2018).
6. World Health Organization, (Website) (2024) WHO Mortality Database.
